# The effectiveness of adjustable trans‐obturator male system (ATOMS) in radiated patients is reduced: A propensity score‐matched analysis

**DOI:** 10.1002/bco2.329

**Published:** 2024-02-11

**Authors:** Javier C. Angulo, Alessandro Giammò, Fabian Queissert, Sandra Schönburg, Carmen González‐Enguita, Andreas Gonsior, Antonio Romero, Francisco E. Martins, Tiago Antunes‐Lopes, Raquel González, Juliusz Szczesniewski, Carlos Téllez, Francisco Cruz, Keith F. Rourke

**Affiliations:** ^1^ Clinical Department, Faculty of Biomedical Science Universidad Europea Madrid Spain; ^2^ Department of Urology Hospital Universitario de Getafe Madrid Spain; ^3^ Department of Neuro‐Urology, CTO/Spinal Cord Unit AOU Città della Salute e della Scienza di Torino Turin Italy; ^4^ Department of Urology and Pediatric Urology University Hospital Muenster Münster Germany; ^5^ Department of Urology and Kidney Transplantation Martin Luther University Halle (Saale) Germany; ^6^ Department of Urology Hospital Fundación Jiménez Díaz Madrid Spain; ^7^ Klinik und Poliklinik für Urologie University of Leipzig Leipzig Germany; ^8^ Department of Urology Hospital Universitario Morales Meseguer Murcia Spain; ^9^ Department of Urology Centro Hospitalar Universitário de Lisboa Norte, Hospital Santa María Lisbon Portugal; ^10^ Department of Urology Centro Hospitalar São João Porto Portugal; ^11^ Faculty of Medicine of Porto I3S Institute Porto Portugal; ^12^ Department of Urology Alberta University, Hospital Edmonton Edmonton Alberta Canada

**Keywords:** adjustable trans‐obturator male system, complications, outcomes, post‐prostatectomy incontinence, radiotherapy, satisfaction

## Abstract

**Objectives:**

This study aimed to compare the effectiveness and safety of the adjustable trans‐obturator male system (ATOMS®) to treat post‐prostatectomy incontinence (PPI) in radiated patients compared with non‐radiated patients, using propensity score‐matching analysis to enhance the validity of the comparison.

**Patients and methods:**

Consecutive men with PPI treated with silicone‐covered scrotal port ATOMS (A.M.I., Feldkirch, Austria) in nine different institutions between 2016 and 2022 were included. Preoperative assessment evaluated 24‐h pad usage, urethroscopy and urodynamics, if indicated. Propensity score‐matching analysis was based on age, length of follow‐up, previous PPI treatment, previous bladder neck stricture, androgen deprivation and pad usage. The primary endpoint was dry rate, defined as no pads post‐operatively with a security pad allowed. The secondary endpoints were complications, device removal and self‐perceived satisfaction with the Patient Global Impression of Improvement (PGI‐I) scale.

**Results:**

Of the 710 included patients, 342 were matched, and the study groups were balanced for the baseline matched variables. The mean baseline 24‐h pad was 4.8 in both groups (*p* = 0.48). The mean follow‐up was 27.5 ± 18.6 months, which was also equivalent between groups (*p* = 0.36). The primary outcome was achieved in 73 (42.7%) radiated patients and in 115 (67.3%) non‐radiated patients (*p* < 0.0001). The mean pad count at the last follow‐up was 1.5 and 0.8, respectively (*p* < 0.0001). There was no significant difference in complications (*p* = 0.94), but surgical revision and device explant rates were higher (*p* = 0.03 and *p* = 0.01, respectively), and the proportion of patients highly satisfied (PGI‐I = 1) was lower in the radiated group (*p* = 0.01). At sensitivity analysis, the study was found to be reasonably robust to hidden bias.

**Conclusion:**

ATOMS implantation significantly outperformed in patients without adjuvant radiation over radiated patients.

## 1 INTRODUCTION

Despite the refinement of prostate cancer care, post‐prostatectomy incontinence (PPI) is still a major sequel to treatment. Therapeutic options must be individualized for each patient according to clinical factors and the patient's characteristics. In recent years, development of new therapeutic alternatives such as male sling techniques has provided a more personalized approach for less severe forms of male stress incontinence after prostatectomy, avoiding the artificial urinary sphincter (AUS) placement in many patients.^1^ As such, a prospective study has confirmed noninferiority between the adjustable trans‐obturator male system (ATOMS) and the AUS, with a higher revision rate for the AUS.^2^

The ATOMS, a male sling with the possibility of post‐operative adjustment, has widened the spectrum of PPI severity that can be treated with the trans‐obturator perineal approach. In this sense, ATOMS is increasingly used to treat mild and moderate PPI in patients with residual sphincteric function.^3^ This device increases urethral resistance by the stretching effect on the urethral wall caused by cushion filling. The compressive action of the bulbar urethra creates a thin capsule around the ATOMS silicone cushion, and this fibrotic capsule causes the stretching effect on the membranous urethra. There is increasing evidence that stretching membranous urethral length can be associated with PPI recovery.^4^ However, the response to an increase in intraurethral pressure depends on urethral rigidity and is not exclusively determined by baseline incontinence severity.^5^

Several prospective and retrospective studies have posed the question of whether radiotherapy is an independent predictor of success for patients treated with ATOMS.^6^, ^7^, ^8^ However, bladder neck contracture and previous urethral surgery, often associated with previous radiation, can also affect continence recovery in these patients.^9^ Additionally, the evolving surgical technique of ATOMS with different generation devices has added difficulty in evaluating the appropriateness of this PPI device in prostate cancer patients treated with radiation.^3^

To date, no randomized comparative study has been developed to evaluate whether ATOMS is an appropriate alternative for patients with PPI after pelvic radiation. We therefore undertook this retrospective multicentre study to compare the efficacy and safety of ATOMS in patients treated with radical prostatectomy with or without radiation using a propensity score‐matching (PSM) analysis to enhance the validity of the comparison.

## 2 PATIENTS AND METHODS

### 2.1 Patient recruitment

After institutional review board (IRB) approval (A08/17), consecutive men with stress predominant PPI who underwent ATOMS implantation between 2016 and 2021 in 11 university hospitals from Europe and Canada were screened for inclusion in this retrospective multicentre study. The effectiveness, safety and self‐reported satisfaction of patients implanted with silicone‐covered scrotal port (SSP) ATOMS after radical prostatectomy with or without radiotherapy were evaluated.

The inclusion criteria were persistent bothersome stress urinary incontinence (SUI) for more than a year after radical prostatectomy. In all cases, the minimum follow‐up after the ATOMS implant was 3 months to allow for post‐operative adjustment. Urinary incontinence not caused by radical prostatectomy and overt neurological disease were exclusion criteria. Baseline severity of incontinence, patient age, previous incontinence device and bladder neck contracture were not exclusion criteria. However, regarding this last criterion, stable urethral patency with a 17‐Ch cystoscope was required. The indication for ATOMS was made by the physician with written informed consent from the patient in every case.

Post‐operative adjustment of the device was performed in the office starting 2–3 weeks after the implantation by percutaneous injection of physiological sodium chloride solution through the SSP membrane and thereafter, when required, at intervals of 3–4 weeks until either dryness was achieved or the maximum filling capacity of the system was reached.

### 2.2 Baseline assessment

All patients were assessed preoperatively with a physical examination including a cough stress test, a bladder diary with 24‐h pad usage, a urethro‐cystoscopy, sonography including post‐void residual volume and urine culture. The definition used to assess PPI severity was based on a 24‐h pad count and classified as mild (1–2 pads), moderate (3–5 pads) and severe (≥6 pads). A baseline 24‐h pad test was also registered, but not in all cases. Urodynamics were performed in cases suggestive of detrusor overactivity and/or excessive residual volume.

### 2.3 PSM analysis

To balance the preoperative characteristics and allow a non‐balanced comparison, a PSM analysis was performed. This is a method of effect estimation used to account for the conditional probability of effect selection, using matched groups of patients who share a similar propensity score and removing confounding bias from observational cohorts where randomization is not possible.^10^ The distribution of measured similar covariates is similar between the subjects in both groups, which allows reducing the bias when comparing them.^11^ Continuous and categorical factors were combined to calculate a propensity score for each patient in the study populations based on the following covariates: age, PPI severity (baseline 24‐h pad count), androgen deprivation, bladder neck stricture, previous incontinence device and length of follow‐up. Patients in the radiated cohort were matched in a 1:1 ratio to patients in the non‐radiated cohort based on the logit of the propensity score.

### 2.4 Outcome measures

Despite the difficulties in universally defining dryness achieved after PPI prosthetic surgery, we used dryness as the primary outcome measure of the study. The dry patient rate was defined as the proportion of patients without pads, although use of a single security pad with occasional and minor urine loss was also allowed. The social continence rate was defined as using one pad/day, regardless of the amount of urine lost.

As PPI severely affects quality of life, we chose a secondary subjective outcome, based on patient‐reported outcome measurement (PROM) with the Patient Global Impression of Improvement (PGI‐I) scale at the last follow‐up compared with the baseline situation (1 [*very much better than before*], 2 [*much better*], 3 [*slightly better*], 4 [*no change*], 5 [*worse*], 6 [*much worse*] and 7 [*very much worse*]). The proportion of patients with the highest satisfaction (PGI‐I = 1) was evaluated. Also, the proportion of patients who considered themselves much better than before (PGI‐I = 1–2) was pooled. The proportion of patients suffering post‐operative complications and the proportion of patients with devices removed during follow‐up were secondary outcomes as well.

### 2.5 Statistical methods

Considering that the dry rate reported in series in which radiated patients predominate was in the range of 39%–75% and 57%–92% in series without predominant radiation,^3^ we assumed a 0.2 proportional difference in success rate for the radiated and non‐radiated cohorts. As a result, 192 matched patients (96 per group) were required to achieve a power of 80% with an alpha error set to 0.05.

To assess the adequacy of the PSM process, the standardized mean difference (SMD) in propensity score between matched patients was calculated, defined by the comparisons of the baseline covariates and of the cumulative distribution functions of the propensity scores of each sample. SMD and *p* values were used to compare outcome variables between cohorts. Meaningful imbalance goes with SMD > 0.1 (10%).^12^

A generalized linear model with a logarithmic link function was built. A sensitivity analysis of the ignorability assumption under PSM was also performed using Rosenbaum's bounding approach to test whether our results were sensitive to such unobserved heterogeneity, with a gamma (Γ) value close to 1 (the higher the Γ, the lower the sensitivity of the study to unmeasured confounders).

Regarding the statistical comparison of measurements, continuous variables were expressed as medians with interquartile ranges (IQRs), and categorical variables were expressed as numbers with percentages. For unmatched samples, Mann–Whitney's and Fisher's exact tests or *χ* ^2^ test were used. For matched samples, differences were evaluated using McNemar's test or paired *t*‐test. All tests were two‐sided, with statistical significance defined as *p* < 0.05. All the statistical analysis was developed using Statistical Analysis System 9.3 (SAS Institute Inc., Cary, NY, USA).

## 3 RESULTS

### 3.1 Matching procedure

**FIGURE 1:**
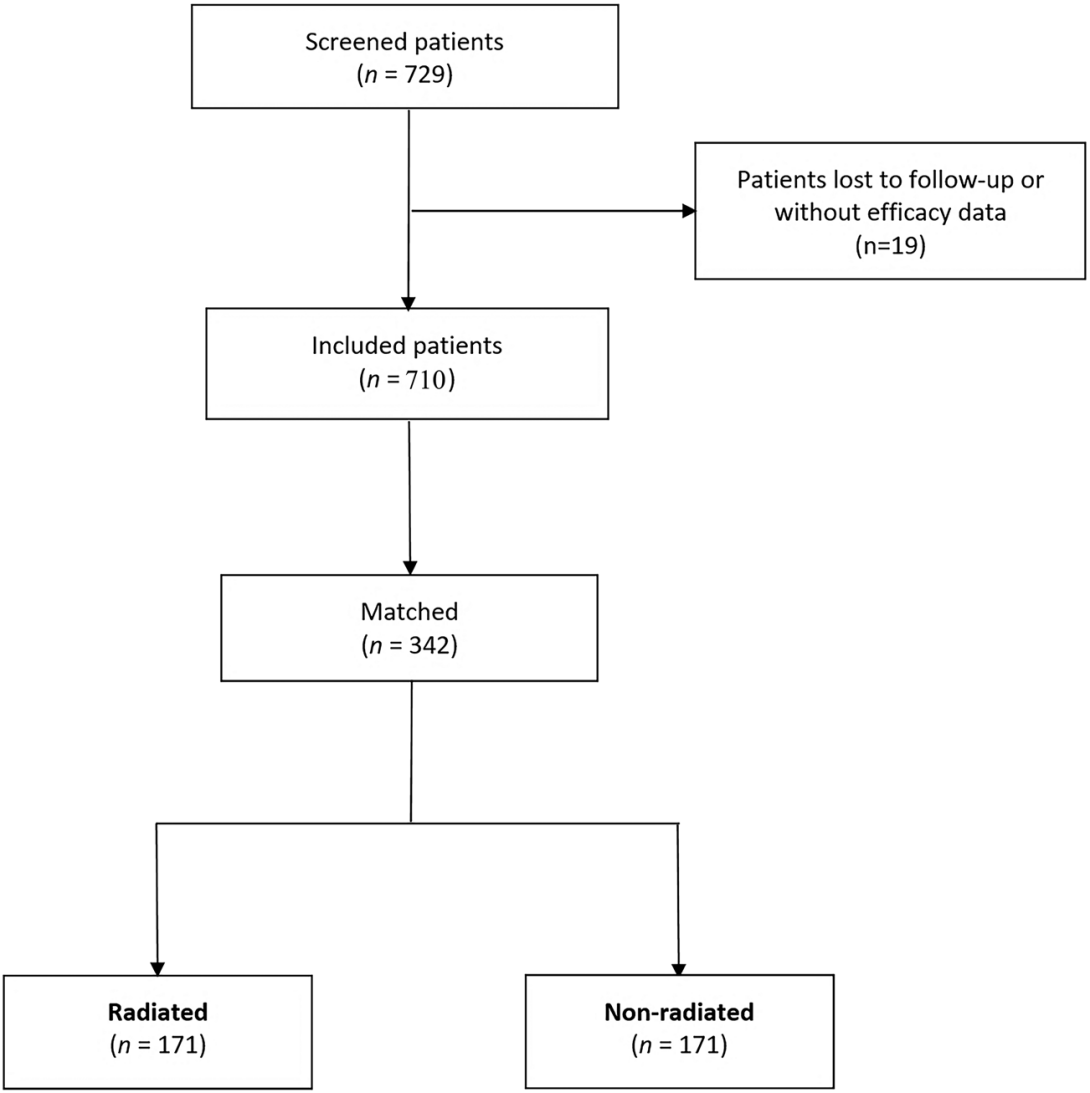
Study flow chart.

**FIGURE 2:**
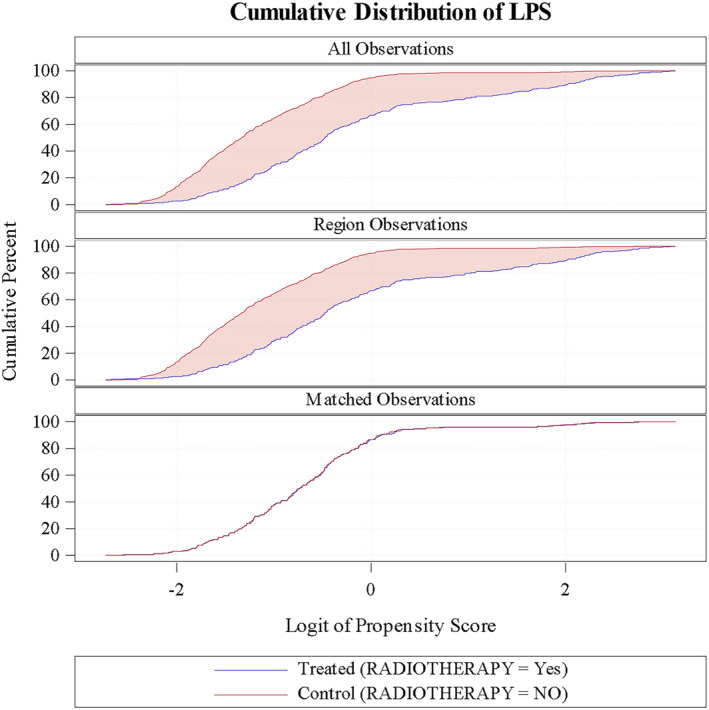
Cumulative distribution of the logit function of propensity score (LPS).

**FIGURE 3:**
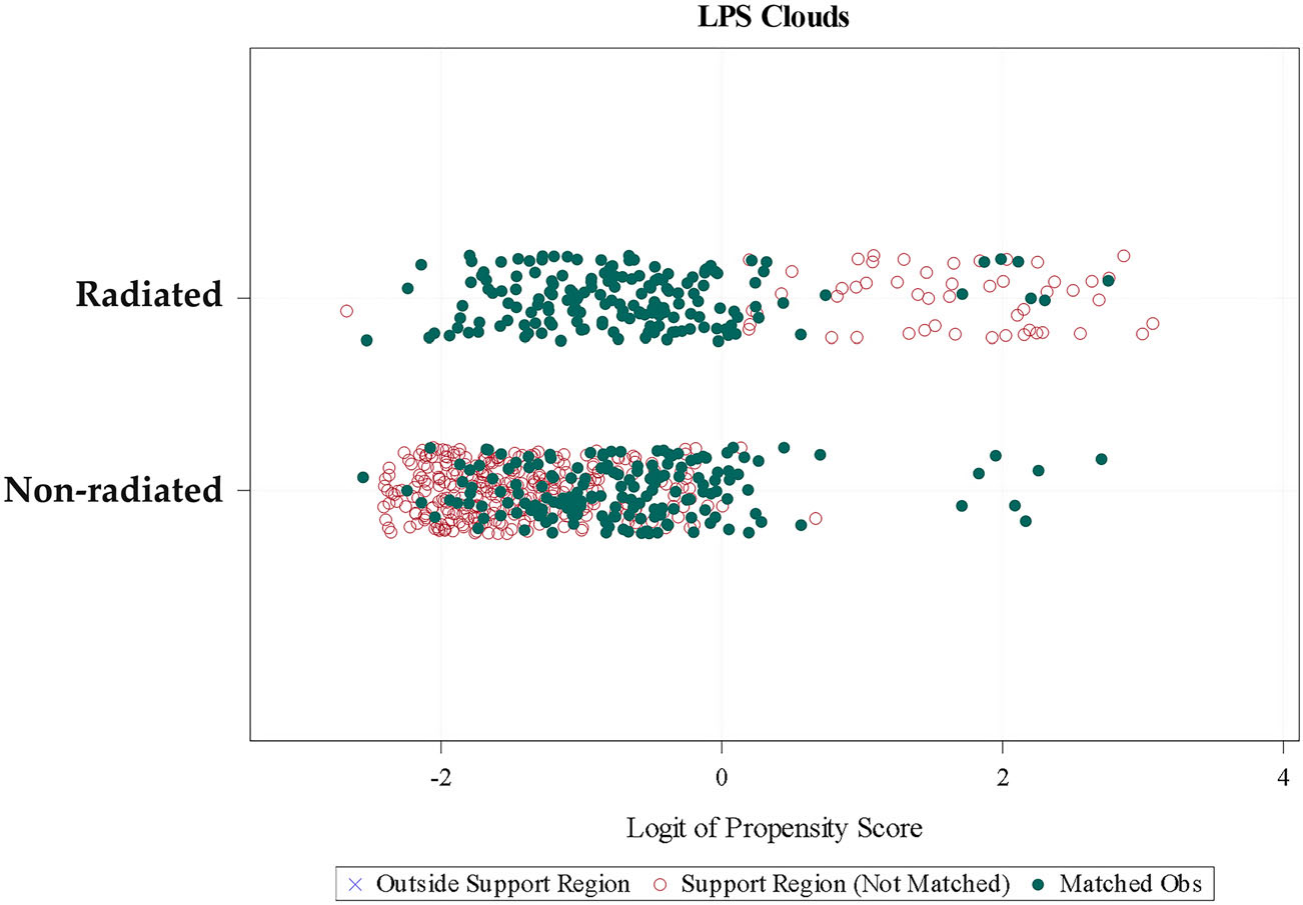
Logit function of propensity score (LPS) clouds with matched observations.

Of the 710 included patients, 342 were matched according to the propensity score (Figure 1). The cumulative distribution function plot of estimated propensity scores (Figure 2) and the logit function of propensity score clouds with matched observations are represented (Figure 3). A satisfactory degree of overlap is confirmed between radiation‐ and non‐radiation‐matched groups.

There were no statistically significant differences between the two cohorts for the variables used for PSM and also for other unmatched baseline variables such as time from radical prostatectomy to ATOMS implant, intraoperative complications and symptoms of overactive bladder (OAB) (Table 1). Equivalence between groups regarding 24‐h pad count (*p* = 0.9) and incontinence severity groups (*p* = 0.48) baseline was confirmed. The mean ± SD of follow‐up was 27.5 ± 18.6 months, 28.3 ± 19 months for radiation and 26.6 ± 18.2 months for non‐radiation (*p* = 0.36).

**TABLE 1:** Comparison of baseline characteristics between groups, radiated and non‐radiated, in the unmatched and matched populations.

	Unmatched population				Matched population			
	Radiated (*n* = 223)	Non‐radiated (*n* = 487)	SMD^a^	*p* value	Radiated (*n* = 171)	Non‐radiated (*n* = 171)	SMD^a^	*p* value
Matched variables								
Age, years (mean ± SD)	71 ± 7	72 ± 8	0.322	0.24	71.3 ± 6.7	71.9 ± 5	0.088	0.58
Previous incontinence surgery, *n* (%)	27 (12.1)	101 (20.7)	0.568	0.005	22 (12.9)	33 (19.3)	0.000	0.1
Androgen deprivation therapy, *n* (%)	46 (20.6)	7 (1.4)	0.857	<0.0001	7 (8.4)	7 (8.4)	0.000	1
Previous bladder neck stricture, *n* (%)	63 (28.2)	85 (17.4)	0.259	0.001	47 (27.5)	49 (28.7)	0.026	0.81
Follow‐up after ATOMS, months (mean ± SD)	37.1 ± 25.2	32.8 ± 22.8	−0317	0.053	28.3 ± 19	26.6 ± 18.2	0.095	0.36
24‐h pad count baseline, pads (mean ± SD)	4 ± 3	4 ± 2	0.234	0.005	4.8 ± 2.2	4.8 ± 2.4	−0.011	0.9
Unmatched variables								
Time since prostatectomy, months (mean ± SD)	59 ± 41.6	71.5 ± 50.5	0.219	0.022	68.1 ± 51.9	64.9 ± 41	0.067	0.84
Intraoperative complications, *n* (%)	2 (0.9)	2 (0.4)	0.101	0.94	1 (1.2)	0 (0)	0.151	1
Overactive bladder symptoms baseline, *n* (%)	27 (12.1)	61 (12.5)	0.095	0.36	16 (19.5)	18 (21.4)	0.047	0.75
Incontinence severity group baseline, *n* (%)								
Mild (1–2 pads/day)	32 (14.4)	74 (15.2)	0.253	0.007	25 (14.6)	19 (11.1)	0.149	0.48
Moderate (3–5 pads/day)	85 (38.1)	240 (49.3)			64 (37.4)	73 (42.7)		
Severe (≥6 pads/day)	106 (47.5)	173 (35.5)			82 (48)	79 (46.2)		

Abbreviations: ATOMS, adjustable trans‐obturator male system; SD, standard deviation; SMD, standardized mean difference.

^a^ An SMD of >0.1 denotes meaningful imbalance in the baseline covariate.

### 3.2 Effectiveness

Dryness was the primary outcome, achieved in 73 (42.7%) radiated patients and in 115 (67.3%) non‐radiated patients (*p* < 0.0001) (Table 2). The mean ± SD 24‐h pad count after adjustment was 1.5 ± 1.6 for radiated patients and 0.8 ± 1.1 for non‐radiated patients (*p* < 0.0001). Accordingly, the number of fillings performed for post‐operative adjustment was also higher in the radiated group than in the non‐radiated group (3.4 ± 2.4 vs. 2.3 ± 2.1; *p* < 0.0001).

**TABLE 2:** Results evaluated in the matched population regarding primary and secondary outcomes.

	Radiated (*n* = 171)	Non‐radiated (*n* = 171)	*p* value
Primary outcomes			
Dryness^a^, *n* (%)	73 (42.7)	115 (67.3)	<0.0001
24‐h pad count after adjustment (mean ± SD)	1.5 ± 1.6	0.8 ± 1.1	<0.0001
Social continence^b^, *n* (%)	106 (62)	143 (83.6)	<0.0001
Residual incontinence severity after adjustment			
Mild (1–2 pads/day), *n* (%)	60 (63.8)	44 (78.6)	<0.0001
Moderate (3–5 pads/day), *n* (%)	30 (31.9)	10 (17.8)	
Severe (≥6 pads/day), *n* (%)	4 (4.3)	2 (3.6)	
Secondary outcomes			
Post‐operative complications (any), *n* (%)	31 (18.1)	30 (17.5)	0.98
Major complications (grade III), *n* (%)	5 (2.9)	4 (2.3)	0.74
OAB symptoms after adjustment, *n* (%)	9 (5.3)	8 (4.7)	0.95
De novo OAB symptoms, *n* (%)	5 (2.9)	3 (1.8)	0.79
Surgical revision, *n* (%)	23 (13.4)	11 (6.4)	0.03
Device explant, *n* (%)	21 (12.3)	8 (4.7)	0.01
PGI‐I very much better (PGI‐I = 1), *n* (%)^c^	32 (38.6)	47 (55.3)	0.03
PGI‐I much better than before (PGI‐I = 1–2), *n* (%)^c^	56 (45.9)	66 (77.6)	0.14

Abbreviations: OAB, overactive bladder; SD, standard deviation.

^a^ Dryness, no pads (with or without security pad with occasional minor urine loss).

^b^ Social continence, 0–1 pads/day.

^c^ Patient Global Impression of Improvement (PGI‐I) was evaluated in 168 cases (83 radiated and 85 non‐radiated).

Social continence was achieved in 106 (62%) patients in the radiation group compared with 143 (83.6%) in patients without radiation (*p* < 0.0001). Regarding the severity of residual incontinence after ATOMS adjustment, a tendency to milder incontinence in patients without radiation is confirmed using the Cochrane–Armitage test (Table 2).

### 3.3 Complications and re‐interventions

During the first 3 months after surgery, post‐operative complications (any grade) developed in 61 (17.8%) patients, 31 (18.1%) radiated and 30 (17.5%) non‐radiated (*p* = 0.98). Table S1 presents the distribution of post‐operative complications in each cohort according to Clavien–Dindo severity categories. There were no grade IV or V complications. Grade III complications were also equivalent between groups, five (2.9%) in radiated patients (perineal haematoma needing drainage, port displacement needing reposition, wound dehiscence, wound infection and perineal pain) and four (2.3%) in patients without radiation (urinary retention needing urethral alignment, perineal pain and scrotal port displacement preventing post‐operative adjustment in two cases) (*p* = 0.74).

Post‐operatively, OAB symptoms were present in 17 patients (5%) after device adjustment. The proportion was equivalent between the radiated and non‐radiated groups (*p* = 0.95) (Table 2). Taking also into account baseline symptoms, ‘de novo’ OAB symptoms were presented in eight cases (2.3%), again without difference between groups (*p* = 0.79) (Table 2).

Surgical revision during follow‐up was performed in 33 cases (9.6%), 22 (12.9%) in the radiated cohort and 11 (6.4%) in the non‐radiated cohort (*p* = 0.03). Table S2 presents all the main reasons for surgical revision in each cohort and their relative frequencies. The main difference is that persistent incontinence was higher in the radiated group with eight radiated cases (4.7%) and only one in the non‐radiated group (0.6%). Consequently, the proportion of patients with explanted devices during follow‐up was higher in the radiated group, 21 cases (12.3%) versus 8 cases (4.7%) (*p* = 0.01; Table 2).

### 3.4 PROMs

PGI‐I at the last follow‐up visit was available in 168 (49.1%) of the matched patients, 83 (48.5%) radiated and 85 (49.7%) non‐radiated. Table S3 presents the distribution of PGI‐I scores in each cohort. The proportion of patients who self‐considered much better compared with baseline (PGI‐I = 1–2) was 45.9% in radiated patients and 77.6% in non‐radiated patients (*p* = 0.14). However, the proportion scoring with the highest satisfaction (very much better; PGI‐I = 1) was 38.6% and 55.3%, respectively (*p* = 0.03) (Table 2).

### 3.5 Sensitivity analysis

The results of the sensitivity analysis for the primary outcome variable are provided in Table S4. The treatment effect turns insignificant at a critical Γ (gamma) value of 0.9. At this inflection point, the *p* value is 0.033, which is greater than the single‐ended type I error (*p* = 0.025). Therefore, the conclusion of the study is inverted if for two individuals k and l in the same paired set, the probability that an individual k is in the radiated group and l in the non‐radiated group is Γ/1 + Γ = 47.3%. If Γ = 1, there is no deviation from the random assignment of each group. In our case, it is 0.9, which is very close to 1.

## 4 DISCUSSION

In the present study, significantly better outcomes were achieved for patients treated with ATOMS exclusively after prostatectomy compared with patients with prostatectomy and adjuvant radiation. Dryness, the primary objective evaluated, was achieved in 43% of patients with radiation compared with 67% of patients without, controlling all the baseline variables that could act as confounding factors. These results go in consonance with previous multicentre studies performed in Spain and Portugal,^7^ Central Europe,^13^ Italy^14^ and Canada,^15^ in which previous radiation was associated with lower continence results.

A secondary objective regarding the proportion of patients who self‐reported being satisfied with the ATOMS device gave interesting results. The proportion of patients with the highest satisfaction (PGI‐I = 1) and the proportion of patients who considered themselves much better than before (PGI‐I = 1–2) were higher in non‐radiated patients, but the difference achieved statistical significance only for PGI‐I = 1 versus the rest.

On the other hand, the post‐operative complications suffered after the ATOMS implant appear equivalent for the radiated and non‐radiated groups. However, the different effectiveness rate implies a difference in surgical revision and the need for secondary treatment, which is twice as high in patients with a history of radiation (13% vs. 6% at a median follow‐up between 2 and 3 years). A recent study has identified factors predictive of failure of ATOMS in patients with PPI and adjuvant radiation, including baseline pad count of >5 pads, need for surgical revision, salvage prostatectomy after failed radiation and bladder neck contracture.^16^

There is a general belief that, regardless of the surgical technique used to correct PPI, previous pelvic irradiation and repeated surgery contribute to worse treatment outcomes.^17^ Our conclusion is firm that ATOMS results outperform in patients without pelvic radiation, and this can be due to the histological changes produced by irradiation in pelvic tissues, which include vascular loss and increased scarring. Global urethral rigidity could partly explain the reduced effectiveness we demonstrated in the radiation cohort. Scarred bulbar and rigid membranous urethra can take place in a patient implanted with ATOMS after radical prostatectomy and adjuvant radiation, even in the absence of bladder neck contracture. In such cases, device adjustment with serial cushion filling may bring a notorious improvement in urine loss, but the patient may not achieve dryness. In non‐radiated patients, the normal elasticity of urethral tissue favours the progressive distribution of pressure to the membranous urethra with cushion filling during adjustment, thus increasing intraurethral pressure and acting as a sphincteric‐reinforcing mechanism.^5^

Chronic radiation‐induced histological changes manifest months to years after exposure and cause atrophy, inflammation, fibrosis and vascular insufficiency.^18^ These changes contribute to the aforementioned increased urethral rigidity and explain that PPI after irradiation is always a challenging situation. Fixed retro‐urethral slings are not recommended for the very high rate of incontinence recurrence due to inadequate urethral closure caused by urethral and periurethral fibrosis.^19^, ^20^ Similarly, the effectiveness of AUS is reduced in radiated patients.^21^ Besides, the histological alterations produced by radiation increase the risk of surgical revision due to urethral atrophy and erosion produced by the circumferential dissection of the urethral bulb for AUS implantation.^22^, ^23^

Irradiated patients may also suffer from the condition termed ‘devastated bladder outlet’, used to describe the combination of stress incontinence and stenosis of the membranous urethra and/or bladder neck. Classical management in these cases includes endoscopic treatment or stricture reconstruction followed by late AUS implantation in the absence of restenosis.^21^ However, in cases with enough residual sphincteric activity, an ATOMS implant can be an alternative that does not predispose to urethral erosion and atrophy.^9^ That makes this adjustable device especially attractive in cases of fragile urethra after a failed former implant, either fixed sling,^24^ AUS^25^ or even repeated ATOMS.^26^ Other reasons to elect ATOMS over an AUS in bad‐profile patients can be reduced manual dexterity or impaired cognitive capacity.^27^

Previous studies with ATOMS have reported that irradiation is a risk factor for complications^28^ and also for the development of de novo OAB symptoms.^29^ However, the present study runs contrary to these observations, probably because the methodology we used allows a better comparison. In observational studies in which randomization is not possible, propensity score methods are used to reduce the bias in estimating treatment effects.^11^ Using PSM, we controlled the risk of higher baseline incontinence in patients with radiation, presented in many previous studies.^7^, ^13^, ^14^, ^15^ Secondarily, the negative influence of concomitant bladder neck contracture was also controlled.^9^ Even other likely confounding factors such as patient age, use of androgen deprivation therapy, previous incontinence treatment and follow‐up were considered in the PSM we used. Finally, only SSP ATOMS generation devices performed in academic centres using ATOMS were included in this study, thus avoiding any confusion raised by former generation devices and also by the learning curve with the device.^3^

Several limitations of the study must be acknowledged. First of all, PSM may not assess and balance all the factors involved in the circumstances of the study, like, for example, diabetes mellitus, smoking and obesity. Nonrecognition could lead to the omission of the effects of several clinically important variables that could affect outcomes. Also, the follow‐up in this series is rather limited, and that implies that late complications leading to device failures may have been underestimated. Finally, PROMs were not registered in half of the patients evaluated, and the findings we reached regarding this secondary outcome may not be totally conclusive. However, the results obtained regarding the multiple outcomes we analysed go in consonance.

## 5 CONCLUSION

In summary, the study shows that ATOMS implantation for PPI significantly outperforms in patients without adjuvant radiation over radiated patients regarding urine loss, PROMs and surgical revision rate. However, despite reduced effectiveness, ATOMS remains an attractive alternative even in a challenging situation, with 43% dryness, 62% social continence and 85% self‐reported satisfaction with the device after radiation. Besides, we confirm that the safety of ATOMS after radiation in terms of post‐operative complications does not differ from that of non‐radiated patients.

## AUTHOR CONTRIBUTIONS

**Javier C. Angulo:** Conceptualization; investigation; data curation; statistical analysis; writing—original draft; writing—review and editing. **Alessandro Giammò:** Conceptualization; investigation; data curation; writing—review and editing. **Fabian Queissert:** Conceptualization; investigation; data curation; writing—review and editing. **Sandra Schönburg:** Conceptualization; investigation; data curation; writing—review and editing. **Carmen González‐Enguita:** Conceptualization; investigation; data curation; writing—review and editing. **Andreas Gonsior:** Conceptualization; investigation; data curation; writing—review and editing. **Antonio Romero:** Conceptualization; investigation; data curation; writing—review and editing. **Francisco E. Martins:** Conceptualization; investigation; data curation; writing—review and editing. **Tiago Antunes‐Lopes:** Conceptualization; investigation; data curation; writing—review and editing. **Raquel González:** Conceptualization; investigation; data curation; writing—review and editing. **Juliusz Szczesniewski:** Conceptualization; investigation; data curation; statistical analysis; writing—review and editing. **Carlos Téllez:** Conceptualization; investigation; data curation; statistical analysis; writing—original draft; writing—review and editing. **Francisco Cruz:** Conceptualization; investigation; data curation; writing—review and editing. **Keith F. Rourke:** Conceptualization; investigation; data curation; writing—review and editing.

## CONFLICT OF INTEREST STATEMENT

The authors declare no conflict of interest associated with the publication of this manuscript.

## Supporting information


**Table S1.** Postoperative complications (Clavien‐Dindo classification) and relative proportions in the matched series and also in each cohort.

**Table S2.** Main reason and relative proportions of surgical revision during follow‐up in the matched series and also in each cohort.

**Table S3.** Patient reported outcomes according to Patient Global Impression of Improvement (PGI‐I) and relative proportions in the matched series and also in each cohort.

**Table S4.** Sensitivity analysis for the primary outcome variable. Gamma and upper bound p‐value for the desired significance level (p < 0.05) marked in orange.

## Data Availability

Full data will be available upon reasonable request from the corresponding author.

## References

[1] Rahnama'i MS , Marcelissen T , Geavlete B , Tutolo M , Hüsch T . Current management of post‐radical prostatectomy urinary incontinence. Front Surg. 2021 Apr 9;8:647656. 10.3389/fsurg.2021.647656 PMID: eCollection 2021. 33898508 PMC8063855

[2] Esquinas C , Ruiz S , de Sancha E , Vazquez M , Dorado JF , Virseda M , et al. Outcomes of a series of patients with post‐prostatectomy incontinence treated with an adjustable transobturator male system or artificial urinary sphincter. Adv Ther. 2021 Jan;38(1):678–690. 10.1007/s12325-020-01563-z 33230712 PMC7854436

[3] Esquinas C , Angulo JC . Effectiveness of adjustable transobturator male system (ATOMS) to treat male stress incontinence: a systematic review and meta‐analysis. Adv Ther. 2019;36(2):426–441. 10.1007/s12325-018-0852-4 30560525 PMC6824356

[4] Oza P , Walker NF , Rottenberg G , MacAskill F , Malde S , Taylor C , et al. Pre‐prostatectomy membranous urethral length as a predictive factor of post prostatectomy incontinence requiring surgical intervention with an artificial urinary sphincter or a male sling. NeurourolUrodyn. 2022;41(4):973–979. 10.1002/nau.24904 PMC9313820 35266177

[5] Ruiz S , Virseda‐Chamorro M , Queissert F , López A , Arance I , Angulo JC . The mode of action of adjustable transobturator male system (ATOMS): intraoperative urethral pressure measurements. Uro. 2021;1:45–53. 10.3390/uro1020007

[6] Angulo JC , Arance I , Esquinas C , Dorado JF , Marcelino JP , Martins FE . Outcome measures of adjustable transobturator male system with pre‐attached scrotal port for male stress urinary incontinence after radical prostatectomy: a prospective study. Adv Ther. 2017 May;34(5):1173–1183. 10.1007/s12325-017-0528-5 28405960

[7] Angulo JC , Cruz F , Esquinas C , Arance I , Manso M , Rodríguez A , et al. Treatment of male stress urinary incontinence with the adjustable transobturator male system: outcomes of a multi‐center Iberian study. NeurourolUrodyn. 2018;37(4):1458–1466. 10.1002/nau.23474 29315765

[8] Angulo JC , Virseda‐Chamorro M , Arance I , Ruiz S , Ojea A , Carballo M , et al. Long‐term outcome of adjustable transobturator male system for stress urinary incontinence in the Iberian multicentre study. NeurourolUrodyn. 2020;39(6):1737–1745. 10.1002/nau.24410 32496606

[9] Ullate A , Arance I , Virseda‐Chamorro M , Ruiz S , Szczesniewski J , Téllez C , et al. ATOMS (adjustable trans‐obturator male system) in patients with post‐prostatectomy incontinence and previously treated urethral stricture or bladder neck contracture. J Clin Med. 2022 Aug 19;11(16):4882. 10.3390/jcm11164882 36013121 PMC9410097

[10] Kane LT , Fang T , Galetta MS , Goyal DKC , Nicholson KJ , Kepler CK , et al. Propensity score matching: a statistical method. Clin Spine Surg. 2020;33(3):120–122. 10.1097/BSD.0000000000000932 31913173

[11] Haukoos JS , Lewis RL . The propensity score. Jama. 2015;314(15):1637–1638. 10.1001/jama.2015.13480 26501539 PMC4866501

[12] Sacco E , Gandi C , Marino F , Totaro A , Di Gianfrancesco L , Palermo G , et al. Artificial urinary sphincter significantly better than fixed sling for moderate post‐prostatectomy stress urinary incontinence: a propensity score‐matched study. BJU Int. 2021;127(2):229–237. 10.1111/bju.15197 32744793

[13] Friedl A , Mühlstädt S , Zachoval R , Giammò A , Kivaranovic D , Rom M , et al. Long‐term outcome of the adjustable transobturator male system (ATOMS): results of a European multicentre study. BJU Int. 2017;119(5):785–792. 10.1111/bju.13684 27868328

[14] Giammò A , Ammirati E , Tullio A , Morgia G , Sandri S , Introini C , et al. Implant of ATOMS® system for the treatment of postoperative male stress urinary incontinence: an Italian multicentric study. Minerva Urol Nefrol. 2020;72(6):770–777. 10.23736/S0393-2249.19.03457-X 31692302

[15] Redmond EJ , Nadeau G , Tu LM , Doiron RC , Steele SS , Herschorn S , et al. Multicentered assessment of clinical outcomes and factors associated with failure of the adjustable transobturator male system (ATOMS). Urology. 2021;148:280–286. 10.1016/j.urology.2020.09.045 33181122

[16] Angulo JC , Téllez C , Giammò A , González‐Enguita C , Schoenburg S , Queissert F , et al. Results of ATOMS (adjustable trans‐obturator male system) in patients with prostate cancer treated with prostatectomy and radiotherapy: a multicenter study. J Clin Med. 2023;12(14):4721. 10.3390/jcm12144721 37510835 PMC10380664

[17] Kim M , Choi D , Hong JH , Kim C‐S , Ahn H , Choo M‐S . Factors contributing to treatment outcomes of post‐prostatectomy incontinence surgery for the selection of the proper surgical procedure for individual patients: a single‐center experience. NeurourolUrodyn. 2018;37(6):1978–1987. 10.1002/nau.23543 29504655

[18] Sterling J , Rahman SN , Varghese A , Angulo JC , Nikolavsky D . Complications after prostate cancer treatment: pathophysiology and repair of post‐radiation urethral stricture disease. J Clin Med. 2023;12(12):3950. 10.3390/jcm12123950 37373644 PMC10299043

[19] Torrey R , Rajeshuni N , Ruel N , Muldrew S , Chan K . Radiation history affects continence outcomes after advance transobturator sling placement in patients with post‐prostatectomy incontinence. Urology. 2013;82(3):713–717. 10.1016/j.urology.2013.03.075 23831073

[20] Wright HC , McGeagh K , Richter LA , Hwang JJ , Venkatesan K , Pysher A , et al. Transobturator sling for post‐prostatectomy incontinence: radiation's effect on efficacy/satisfaction. Can J Urol. 2017;24:8998–9002. 28971786

[21] Guillaumier S , Solomon E , Jenks J , Pakzad M , Hamid R , Ockrim J , et al. Radiotherapy is associated with reduced continence outcomes following implantation of the artificial urinary sphincter in men with post‐radical prostatectomy incontinence. Urol Ann. 2017;9(3):253–256. 10.4103/UA.UA_25_17 28794592 PMC5532893

[22] Manunta A , Guillé F , Patard JJ , Lobel B . Artificial sphincter insertion after radiotherapy: is it worthwhile? BJU Int. 2000;85(4):490–492. 10.1046/j.1464-410x.2000.00484.x 10691830

[23] Mamane J , Sanchez S , Lellouch AG , Gaillard V , Poussot B , Tricard T , et al. Impact of radiation therapy on artificial urinary sphincter implantation in male patients: a multicenter study. NeurourolUrodyn. 2022;41(1):332–339. 10.1002/nau.24825 34816473

[24] Queissert F , Rourke K , Schönburg S , Giammò A , Gonsior A , González‐Enguita C , et al. ATOMS (adjustable transobturator male system) is an effective and safe second‐line treatment option for recurrent urinary incontinence after implantation of an AdVance/AdVance XP fixed male sling? A multicenter cohort analysis. J Clin Med. 2021;11(1):81. 10.3390/jcm11010081 35011821 PMC8745557

[25] Angulo JC , Esquinas C , Arance I , Rodríguez A , Pereira J , Rabassa M , et al. Adjustable transobturator male system after failed surgical devices for male stress urinary incontinence: a feasibility study. Urol Int. 2018;101(1):106–113. 10.1159/000489316 29953998

[26] Angulo JC , Schönburg S , Giammò A , Queissert F , Gonsior A , González‐Enguita C , et al. Artificial urinary sphincter or a second adjustable transobturator male system offer equivalent outcomes in patients whom required revision on the initial ATOMS device: an international multi‐institutional experience. NeurourolUrodyn. 2021;40(3):897–909. 10.1002/nau.24646 33645867

[27] Comiter CV . Surgery insight: surgical management of postprostatectomy incontinence—the artificial urinary sphincter and male sling. Nat Clin Pract Urol. 2007;4(11):615–624. 10.1038/ncpuro0935 17982438

[28] Mühlstädt S , Angulo JC , Mohammed N , Schumann A , Fornara P . Complications of the urinary incontinence system ATOMS: description of risk factors and how to prevent these pitfalls. World J Urol. 2020;38(7):1795–1803. 10.1007/s00345-019-02962-w 31542824

[29] Schönburg S , Bauer W , Mohammed N , Brössner C , Fornara P . De novo OAB after ATOMS: an underestimated problem or a rare side effect? Front Surg. 2019;6:72. 10.3389/fsurg.2019.00072 31921886 PMC6928117

