# Supplementary material for: The effectiveness of adjustable trans‐obturator male system (ATOMS) in radiated patients is reduced: A propensity score‐matched analysis
Source: BJUI Compass. 2024 Feb 11;5(4):506–14. doi: 10.1002/bco2.329 (PMC11019248; doi:10.1002/bco2.329)
Supplement: Supplementary file 1 — Table S1. Postoperative complications (Clavien‐Dindo classification) and relative proportions in the matched series and also in each cohort. [file BCO2-5-506-s003.docx]

**Table S1.** Postoperative complications (Clavien-Dindo classification) and relative proportions in the matched series and also in each cohort.

|  | **Radiated** | **Non-radiated** | **Total** |
| --- | --- | --- | --- |
| **Postoperative complications** | | | |
| No complications, n (%) | 140 (81.9) | 141 (82.5) | 281 (82.2) |
| Grade I, n (%) | 16 (9.4) | 15 (8.8) | 31 (9.1) |
| Grade II, n (%) | 10 (5.8) | 11 (6.4) | 21 (6.1) |
| Grade III, n (%) | 5 (2.9) | 4 (2.3) | 9 (2.6) |
| Total | 171 (100) | 171 (100) | 342 (100) |
