# Supplementary material for: The effectiveness of adjustable trans‐obturator male system (ATOMS) in radiated patients is reduced: A propensity score‐matched analysis
Source: BJUI Compass. 2024 Feb 11;5(4):506–14. doi: 10.1002/bco2.329 (PMC11019248; doi:10.1002/bco2.329)
Supplement: Supplementary file 2 — Table S2. Main reason and relative proportions of surgical revision during follow‐up in the matched series and also in each cohort. [file BCO2-5-506-s001.docx]

**Table S2.** Main reason and relative proportions of surgical revision during follow-up in the matched series and also in each cohort.

|  | **Radiated** | **Non-radiated** | **Total** |
| --- | --- | --- | --- |
| **Surgical revision during follow-up** | | | |
| Persistent incontinence | 8 (36.4) | 1 (9.1) | 9 (27.3) |
| Skin erosion by scrotal port | 4 (18.2) | 3 (27.3) | 7 (21.2) |
| Perineal pain | 4 (18.2) | 2 (18.2) | 6 (18.2) |
| Infection of the device | 4 (18.2) | 2 (18.2) | 6 (18.2) |
| Port displacement needing reposition | 1 (4.5) | 2 (18.2) | 3 (9.1) |
| Wound dehiscence | 1 (4.5) | - | 1 (3) |
| Perineal hematoma | - | 1 (9.1) | 1 (3) |
| Total | 22 (100) | 11 (100) | 33 (100) |
