# Supplementary material for: The effectiveness of adjustable trans‐obturator male system (ATOMS) in radiated patients is reduced: A propensity score‐matched analysis
Source: BJUI Compass. 2024 Feb 11;5(4):506–14. doi: 10.1002/bco2.329 (PMC11019248; doi:10.1002/bco2.329)
Supplement: Supplementary file 3 — Table S3. Patient reported outcomes according to Patient Global Impression of Improvement (PGI‐I) and relative proportions in the matched series and also in each cohort. [file BCO2-5-506-s004.docx]

**Table S3.** Patient reported outcomes according to Patient Global Impression of Improvement (PGI-I) and relative proportions in the matched series and also in each cohort.

|  | **Radiated** | **Non-radiated** | **Total** |
| --- | --- | --- | --- |
| **Patient Global Impression of Improvement** | | | |
| PGI-I=1, very much better | 32 (38.6) | 47 (55.3) | 79 (47) |
| PGI-I=2, much better | 24 (28.9) | 19 (22.3) | 43 (25.6) |
| PGI-I=3, slightly better | 15 (18.1) | 16 (18.8) | 31 (18.4) |
| PGI-I=4, same as before | 8 (9.6) | 2 (2.4) | 10 (6) |
| PGI-I=5, worse | 3 (3.6) | - | 3 (1.8) |
| PGI-I=6, much worse | 1 (1.2) | 1 (1.2) | 2 (1.2) |
| PGI-I=7, very much worse | - | - | - |
| Total ^#^ | 83 (100) | 85 (100) | 168 (100) |

^#^ Evaluated in 168 cases.
