# Supplementary material for: The effectiveness of adjustable trans‐obturator male system (ATOMS) in radiated patients is reduced: A propensity score‐matched analysis
Source: BJUI Compass. 2024 Feb 11;5(4):506–14. doi: 10.1002/bco2.329 (PMC11019248; doi:10.1002/bco2.329)
Supplement: Supplementary file 4 — Table S4. Sensitivity analysis for the primary outcome variable. Gamma and upper bound p‐value for the desired significance level (p < 0.05) marked in orange. [file BCO2-5-506-s002.docx]

**Table S4.** Sensitivity analysis for the primary outcome variable. Gamma and upper bound p-value for the desired significance level (p<0.05) marked in orange.

| **Gamma** | **Mean** | **Variance** | **T test** | **P value** |
| --- | --- | --- | --- | --- |
| 0.50 | 4902.00 | 373641.33 | 5.332 | 0.000 |
| 0.55 | 5218.26 | 384916.67 | 4.744 | 0.000 |
| 0.60 | 5514.75 | 394074.84 | 4.216 | 0.000 |
| 0.65 | 5793.27 | 401432.84 | 3.738 | 0.000 |
| 0.70 | 6055.41 | 407256.12 | 3.300 | 0.001 |
| 0.75 | 6302.57 | 411768.00 | 2.897 | 0.002 |
| 0.80 | 6536.00 | 415157.04 | 2.523 | 0.006 |
| 0.85 | 6756.81 | 417583.08 | 2.174 | 0.016 |
| **0.90** | **6966.00** | **419182.11** | **1.846** | **0.033** |
| 0.95 | 7164.46 | 420070.14 | 1.538 | 0.063 |
| 1.00 | 7353.00 | 420346.50 | 1.247 | 0.107 |
| 1.05 | 7532.34 | 420096.44 | 0.971 | 0.167 |
| 1.10 | 7703.14 | 419393.33 | 0.708 | 0.240 |
| 1.15 | 7866.00 | 418300.47 | 0.457 | 0.324 |
| 1.20 | 8021.45 | 416872.56 | 0.217 | 0.414 |
| 1.25 | 8170.00 | 415157.04 | -0.013 | 0.505 |
| 1.30 | 8312.09 | 413195.05 | -0.234 | 0.592 |
| 1.35 | 8448.13 | 411022.38 | -0.447 | 0.672 |
| 1.40 | 8578.50 | 408670.21 | -0.652 | 0.742 |
| 1.45 | 8703.55 | 406165.71 | -0.851 | 0.802 |
| 1.50 | 8823.60 | 403532.64 | -1.042 | 0.850 |
